# Supplementary material for: Fronto-thalamic networks and the left ventral thalamic nuclei play a key role in aphasia after thalamic stroke
Source: Commun Biol. 2024 Jun 7;7:700. doi: 10.1038/s42003-024-06399-9 (PMC11161613; doi:10.1038/s42003-024-06399-9)
Supplement: Supplementary file 2 — Supplementary Information [file 42003_2024_6399_MOESM2_ESM.pdf]

# Supplementary Information: Fronto-thalamic networks and the left ventral thalamic nuclei: key players in aphasia after thalamic stroke

Ida Rangus,[1,2,†*] Ana Sofia Rios,[1,†] Andreas Horn,[1,3,4,5,6] Merve Fritsch,[7] Ahmed Khalil,[1,2] Kersten Villringer,[1,2] Birgit Udke,[8] Manuela Ihrke,[8] Ulrike Grittner,[9,10] Ivana Galinovic,[1,2] Bassam Al-Fatly,[3] Matthias Endres,[1,2,10,11,12,13] Anna Kufner,[1,2,•] and Christian H. Nolte[1,2,10,11,•]

**[†]These authors contributed equally.**

**[•]These authors jointly supervised this work.**

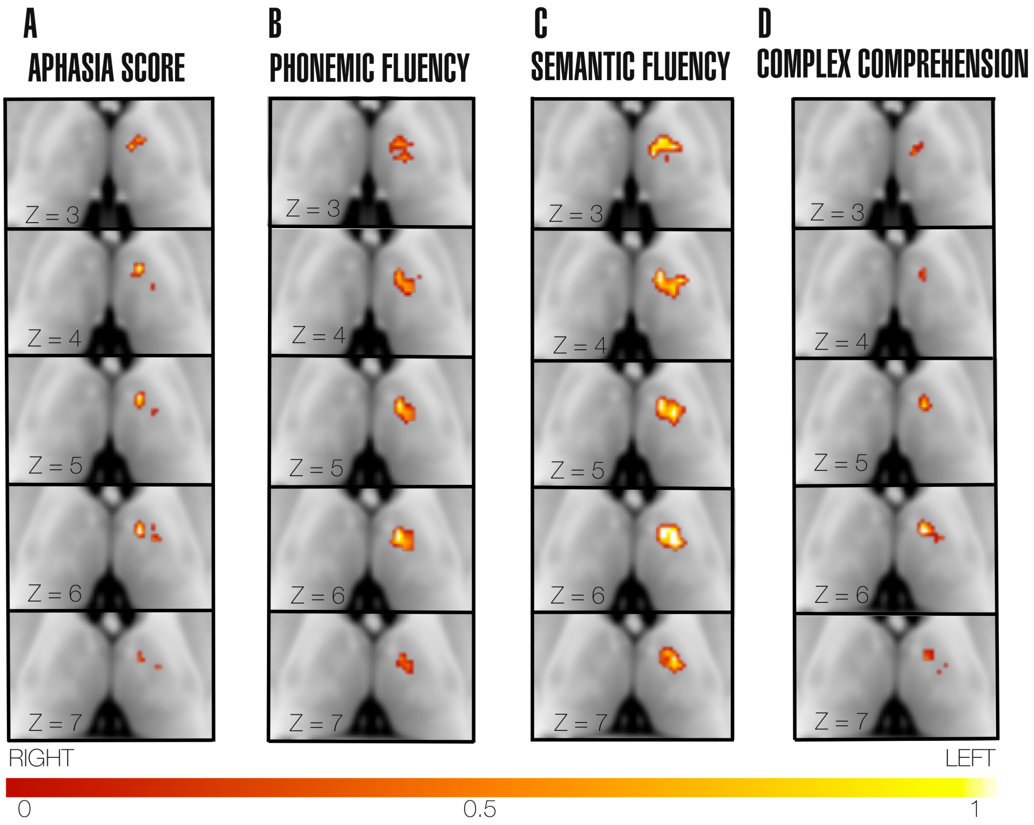


**Figure 1. Resulting regions of interest (ROIs) of the multivariate lesion symptom mapping analysis** overlaid onto coronal view of a brain template, showing (A) general language impairments using aphasia score, and significant subdomain-specific impairments: (B) phonemic fluency, (C) semantic fluency and (D) complex comprehension.

**Table 1:** Comparison of the frequency of aphasia and aphasia scores in patients with thalamic stroke on the left vs. right side.

| | **Left-sided thalamic stroke** | **Right-sided thalamic stroke** | ***P-value*** |
|---|---|---|---|
| **No. of individuals with aphasia (%)** | 21 (46%)<br>(n = 46) | 13 (33%)<br>(n = 39) | 0.274[a] |
| **Aphasia score Mean (±SD)** | 129.0 (±15.1)<br>(n = 40) | 136.1 (±8.3)<br>(n = 36) | **0.043[b]** |

[a]Chi-squared test, [b]Mann-Whitney U-Test

**Table 2: Results from subdomain-specific language tasks.** The numbers indicate how many patients scored in the given category in each subdomain.

| | **Language subdomains (n = 85)** | | | | | | | |
|---|---|---|---|---|---|---|---|---|
| **Results** | **simple comprehension** | **complex comprehension** | **phonemic fluency** | **semantic fluency** | **naming** | **reading** | **writing** | **repeating** |
| no disorder | 70 | 43 | 34 | 40 | 70 | 80 | 80 | 84 |
| mild disorder | 10 | 29 | 19 | 15 | 7 | 5 | 4 | 0 |
| moderate disorder | 0 | 13 | 27 | 24 | 4 | 0 | 1 | 1 |
| severe disorder | 5 | 0 | 5 | 6 | 4 | 0 | 0 | 0 |

**Table 3:** Complete overview of the results of the multivariate lesion symptom mapping analysis showing number of supratresholded voxels, *r*-values und *P*-values.

| **Analysis** | **Suprathresholded voxels (2x2x2 mm)** | ***r*-value** | ***P*-value** | ***Optimal sparseness*** |
|---|---|---|---|---|
| *GENERAL LANGUAGE ASSESSMENT*<br>**Aphasia score** | **24** | **0.495** | **<0.001** | **-0.242** |
| *SUBDOMAINS* | | | | |
| Simple comprehension | 0 | 0.192 | 0.078 | -0.232 |
| **Complex comprehension** | **25** | **0.383** | **<0.001** | **0.457** |
| **Phonemic fluency** | **42** | **0.482** | **<0.001** | **0.475** |
| **Semantic fluency** | **47** | **0.537** | **<0.001** | **0.475** |
| Naming | 0 | 0.477 | <0.001* | -0.212 |
| Reading | 0 | 0.081 | 0.463 | -0.212 |
| Writing | 0 | 0.230 | 0.346 | 0.387 |
| Repeating | 0 | 0.076 | 0.512 | 0.232 |

*significant *P*-value with zero thresholded voxels

**Table 4:** Cluster analysis of ROIs from LSM using Mango Software

| Analysis | Cluster size (mm3) | *Peak Coordinates* | *Central Coordinates* |
|---|---|---|---|
| **Aphasia score** | **67** | **X = -8**<br>**Y = -8**<br>**Z= 4** | **X = -8**<br>**Y = -8**<br>**Z = 5** |
| | 18 | X = -14<br>Y= -12<br>Z= 8 | X = -14<br>Y = -12<br>Z = 7 |
| | 2 | X =-12<br>Y= -8<br>Z=6 | X = -12<br>Y = -8<br>Z = 6 |
| **Subdomains** | | | |
| Complex comprehension | **72** | **X = -8**<br>**Y = -10**<br>**Z = 6** | **X = -9**<br>**Y = -9**<br>**Z = 6** |
| | 2 | X = -12<br>Y = -14<br>Z = 8 | X = -12<br>Y = -14<br>Z = 8 |
| Phonemic fluency | **190** | **X = -8**<br>**Y = -10**<br>**Z = 6** | **X = -10**<br>**Y = -11**<br>**Z = 5** |
| Semantic fluency | **340** | **X = -8**<br>**Y = -8**<br>**Z = 4** | **X = -11**<br>**Y = -10**<br>**Z = 5** |

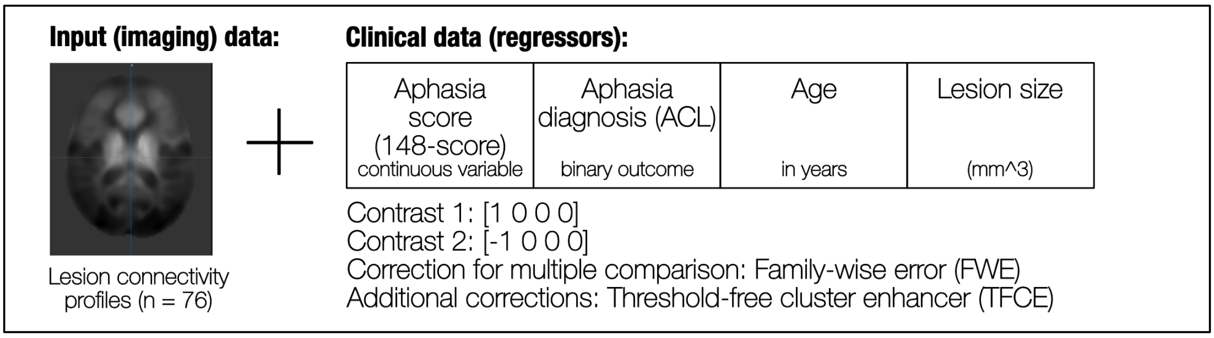


**Figure 2. Non-parametric analysis set up.** Data input for permutation analysis, based on the General Linear Model. Two contrasts were established on aphasia scores to extract brain regions most strongly connected to thalamic lesions from patients with higher aphasia scores (more severe language deficits) with contrast 1, and brain regions most connected to thalamic lesions from patients with lower aphasia scores (less severe to no language deficits) with contrast 2.

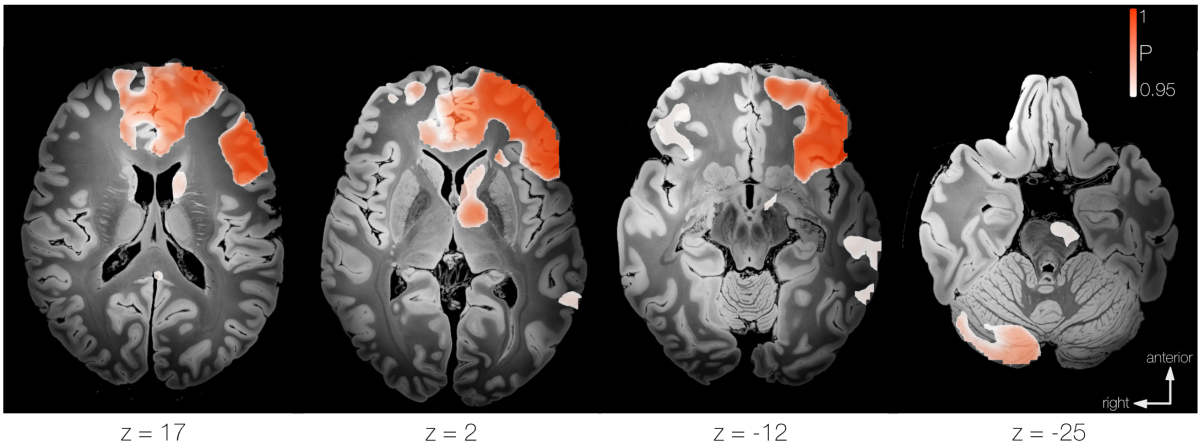


**Figure 3. Non-parametric analysis excluding binary outcome.** Thalamic aphasia network including only aphasia scores as variable of interest, and lesion size and age as covariates. FWE-corrected map thresholded at $P < 0.05$, first contrast (worse language performance) is shown in white-red.

**Table 5.** Structures and regions significantly connected to thalamic lesions associated with worse language performance.

| Map / Atlas | Harvard-Oxford Cortical Structures | Harvard-Oxford Subcortical Structures | JHU White Matter Tractography Atlas | Atlas of the Human Cerebellum |
|---|---|---|---|---|
| Correlations map (FDR $\alpha < 0.05$) | Frontal Pole<br>Insular Cortex<br>Superior Frontal Gyrus<br>Middle Frontal Gyrus<br>Inferior Frontal Gyrus, pars triangularis<br>Inferior Frontal Gyrus, pars opercularis<br>Precentral Gyrus<br>Middle Temporal Gyrus, posterior division and temporooccipital part<br>Superior Parietal Lobule<br>Supramarginal Gyrus, posterior division<br>Angular Gyrus<br>Lateral Occipital Cortex, superior division<br>Paracingulate Gyrus<br>Cingulate Gyrus, anterior division<br>Cingulate Gyrus, posterior division<br>Precuneous Cortex<br>Frontal Orbital Cortex<br>Parahippocampal Gyrus, anterior division<br>Frontal Operculum Cortex | Left Caudate<br>Left Putamen<br>Left globus pallidus<br>Brainstem | Anterior thalamic radiation<br>Corticospinal tract<br>Left Cingulum (cingulate gyrus)<br>Forceps minor<br>Inferior fronto-occipital fasciculus<br>Left Superior longitudinal fasciculus<br>Left Uncinate fasciculus | Crus I<br>Crus II |
| Thalamic aphasia network (pFWE < 0.05) | Left Frontal Pole<br>Left Insular Cortex<br>Left Superior Frontal Gyrus<br>Left Middle Frontal Gyrus<br>Left Inferior Frontal Gyrus, pars triangularis<br>Left Inferior Frontal Gyrus, pars opercularis<br>Left Precentral Gyrus<br>Left Frontal Medial Cortex<br>Left Juxtapositional Lobule Cortex (formerly Supplementary Motor Cortex)<br>Left Paracingulate Gyrus<br>Left Cingulate Gyrus, anterior division<br>Left Frontal Orbital Cortex<br>Left Frontal Operculum Cortex | Left globus pallidus | Left Anterior thalamic radiation<br>Left Cingulum (cingulate gyrus)<br>Forceps minor<br>Left Inferior fronto-occipital fasciculus<br>Left Superior longitudinal fasciculus<br>Left Uncinate fasciculus | |
